# Neurosurgical complications based on modified Clavien-Dindo grade and its correlation with modified Rankin scale: a prospective cohort study from Nepal

**DOI:** 10.1097/MS9.0000000000004854

**Published:** 2026-03-26

**Authors:** Milan K.C., Susmin Karki, Gopal Sedain

**Affiliations:** a Department of Surgery, Tribhuvan University Institute of Medicine, Maharajgunj, Nepal; b Maharajgunj Medical Campus, Tribhuvan University Institute of Medicine, Maharajgunj, Nepal; c Department of Neurosurgery, Tribhuvan University Institute of Medicine, Maharajgunj, Nepal

**Keywords:** Nepal, outcome prediction, postoperative complications, prospective cohort study

## Abstract

**Background:**

The Clavien-Dindo classification (CDC) is a standardized system for classifying surgical complications. The severity of a complication is graded based on the type of therapy required to treat the complication. The assessment of surgical complications is an important tool in neurosurgical practice because it can improve the safety and quality of patient treatment. Among various outcome scales for patient functional performance, the modified Rankin scale (mRS) is a widely accepted outcome scale.

**Methods:**

A prospective cohort study was conducted over a 12-month period. Postoperative complications were graded according to the Modified Clavien-Dindo (MCD) grading. Daily evaluation of the patient was conducted during their hospital stay, and they were followed up until postoperative day 30. The outcome was graded as per the mRS. The correlation between MCD grade and the mRS was analyzed. Statistical analysis was performed with SPSS version 25.

**Results:**

The study included a total of 180 patients. The maximum number of patients was from 41 to 60 year age group, with 103 (57.2%) being male. A total of 82% of surgeries were cranial, and 62% of patients underwent elective surgery. The mean hospital length of stay was 8.78 days (SD ± 5.2). Overall, 44% of the patients had postoperative complications with a mortality rate of 4.4%. The patients with a higher grade of MCD had a bad outcome, as measured by mRS, with a statistically significant *P*-value (<0.05).

**Conclusion:**

The severity of the post-operative complications, graded as per MCDG, correlated with patient outcomes measured according to the mRS.

## Introduction

Like any surgical discipline, neurosurgery also entails complications and inherits risk considerably higher than other surgical specialties. Surgical complication is defined as “any deviation from the ideal postoperative course that is not inherent in the procedure and does not comprise a failure to cure”[1]. Assessment of surgical complications is an important tool in neurosurgical practice because it can improve the safety and quality of patient treatment. The Clavien-Dindo Classification (CDC) is a standardized system for the registration of surgical complications. The classification was initially developed by Clavien in 1992 to report negative outcomes after cholecystectomy[2]. It was modified by Dindo *et al* in 2004 to increase its accuracy and acceptability in clinical practice. The major characteristic of the CDC system is that the severity of a complication is graded based on the type of therapy required to treat the complication[3]. The CDC system has been validated and accepted worldwide for use in many fields of surgery. To enhance patient outcomes, it is crucial to fully report and objectively analyze treatment-related problems, ideally prospectively. In this initial classification and its later derivatives, complications are differentiated from failure to cure and sequelae. The CDC defines a complication as any deviation from the normal postoperative course. Some of the postoperative complications following neurosurgical procedures are postoperative hemorrhage, infections, postoperative hydrocephalus, deep vein thrombosis, cerebrospinal fluid (CSF) leak, and so on.

HIGHLIGHTS

- Modified Clavien Dindo grade is a predictive tool to measure the postoperative outcome as per the modified Rankin scale in neurosurgical patients.
- The severity of the post-operative complications graded as per modified Clavein Dindo grading correlated with patient outcomes measured according to the modified Rankin scale.
- Proper stratification of postoperative complications in neurosurgery helps predict postoperative patient outcomes.

Various outcome scales for patient functional performance are used, including the modified Rankin scale (mRS), which is used in this study (Table 1). In this study, postoperative complications were graded according to the Modified Clavien-Dindo (MCD) grade, and the MCD grade was correlated with the mRS. The incidence of neurosurgical complications is referenced from various studies. One of the major studies, a multicenter prospective observational study on postoperative neurological complications after cranial surgery by Venkatapura *et al*, included 279 cases (institution 1: 110, institution 2: 100, and institution 3: 69). The total number of neurological complications was 53 (19%)[4]. Another study by Jennifer E Fugate showed that overall complication rate of neurosurgical procedures was approximately 14%[5]. This cohort study has been reported in line with the STROCSS 2025 guidelines[6].

**Table 1:** Clavien–Dindo classification for neurosurgical complications: Description of the modified grading system used to categorize postoperative complications by severity.

Classification of neurosurgical complication	
Grade I	Any non-life-threatening deviation from normal postoperative course, not requiring invasive treatment
Grade Ia	Complication requiring no drug treatment
Grade Ib	Complication requiring drug treatment
Grade II	Complication requiring invasive treatment such as surgical, endoscopic, or endo vascular interventions
Grade IIa	Complication requiring intervention without general anesthesia
Grade IIb	Complication requiring intervention with general anesthesia
Grade III	Life threatening complications requiring management in ICU
Grade IIIa	Complication involving single organ failure
Grade IIIb	Complication involving multiple organ failure
Grade IV	Complication resulting death

## Materials and methods

This is a quantitative, prospective, analytical study conducted at the Department of Neurosurgery. All patients admitted to the Department of Neurosurgery and undergoing operative procedures were eligible to participate in the study. A nonprobability sampling method was utilized to select the required sample.

### Sample size

All eligible cases are received during the study period. The sample size is calculated based on the overall complications and prevalence. The expected prevalence is 19% with a 95% CI, 80% power, and a permissible error of 6%.

Prevalence of complications following neurosurgery (*p*) = 19%[7]

*q* = 100–19% = 81%

Z at 95% CI = 1.96

Maximum Permissible Error (*d*) = 6%

*n* = z^2^pq/d^2^

= 164

drop out of 10%

Thus, the required sample size is 180.

**Inclusion criteria**:

All operated neurosurgical cases.

**Exclusion criteria**:

Patients/caregivers not giving consent.

Patients who could not follow up till postoperative day 30.

Patient with a pre-existing disability.

Patients who underwent conservative management.

### Research methodology

This is a quantitative, analytical, longitudinal, and prospective study that includes all patients undergoing neurosurgery during the study period. The study was conducted over a 1-year period, following Institutional Review Committee (IRC) approval, from August 2022 to August 2023.

#### Tools and techniques for data collection

In this research, a self-designed proforma was used as a tool. The proforma was designed in accordance with the research title by the principal investigator, following discussions with the guide and co-guide. All the operated neurosurgical cases were included in the sample. The patient was followed in the postoperative period till postoperative day 30, and postoperative complications were classified if they occurred. At the end, the MCD Grade of neurosurgical complications was correlated with the mRS. The research was conducted after approval of the research proposal by the Department of Neurosurgery and the IRC of the Research Department. Data entered and analyzed in SPSS 25.

#### Management protocol of patients

All patients who underwent neurosurgery were recruited for the study after taking informed consent. Demographic data, detailed history, physical examination, and pre-operative parameters were recorded in the standard proforma. Daily evaluation of the patient during the hospital stay was conducted, and any complications (if present) were noted, graded according to the Clavien-Dindo Grading system, and managed in accordance with institutional practice. Every patient who undergoes neurosurgical treatment is transferred to the intensive care unit (ICU), regardless of complications. However, only patients who remain in the ICU for more than 24 hours are classified as Grade 3 by the CDC. The patients were followed in the postoperative period until postoperative day 30, and postoperative complications were classified if any occurred. The patient’s outcome was measured using the mRS. The endpoint of the study was in-hospital mortality or follow-up of patients till postoperative day 30 (either in person or through telephone after discharge). Complications were classified as mild (MCD I), moderate (MCD II), and severe (MCD III and IV)[8]. The primary outcome measure was the mRS (mRS score) at 1 month. A score of 0–3 was considered a good outcome, and 4–6 was considered a bad outcome[9].

#### Statistical analysis

Statistical analysis was performed with SPSS v. 25. Results were expressed as mean ± standard deviation, median, and range wherever applicable. Student’s *t*-test was applied for continuous variables expressed as mean ± standard deviation, and the Chi-square test was applied for categorical variables shown as count and percentage. The association between MCD grade and mRS outcome was evaluated using the Chi-square test and linear-by-linear association for trend. Differences in hospital length of stay (LOS) between patients with and without complications were compared using the independent-samples *t*-test. For comparisons across MCD severity groups (mild, moderate, and severe), mean values were reported. The confidence interval of 95% was taken, and a *P*-value < 0.05 was considered statistically significant.

## Results

The study included a total of 180 patients.

### Demographic profile

#### Age-wise distribution of complications

The maximum number of patients was from the 41–60-year age group. The mean age of the patients was 39.88 (SD ± 22.4; Table 2).

**Table 2:** Age-wise distribution of postoperative complications: Distribution of neurosurgical patients by age group and presence or absence of complications.

Age group	Complications		
	Yes	No	Total
<20 years	17	25	42
21–40	17	20	37
41–60	27	32	59
61–80	18	23	41
>81	1	0	1
Total	80	100	180

#### Gender-wise distribution of complications

Out of 180 patients, 77 (43%) were female, and 103 (57%) were male. Thirty-nine females and 41 males developed complications.

#### Frequency of patients with comorbidities

Out of 80 patients who had developed complications, 28 patients had pre-existing hypertension, 10 had diabetes, 5 had hypothyroidism, 2 had atrial fibrillation, and the other 3 had rheumatic heart disease, coronary artery disease, and asthma, each in a single patient.

#### Type of surgery

Out of 180, 147(82%) were cranial and 33 (18%) were spinal surgery, 112 (62%) were elective, and 68 (38%) were emergency surgeries.

#### Different categories of diagnosis

Out of 180 patients, 15 were congenital cases, 54 had neoplasms, and so on, as depicted in the table below. The cases were categorized as either cranial or spinal (Table 3).

**Table 3:** Diagnostic categories of operated neurosurgical patients: Classification of all neurosurgical cases into cranial and spinal diagnostic groups with their frequencies.

Types of diagnosis	Type of case		Total
	Cranial	Spinal	
Congenital	11	4	15
Neoplasm	50	4	54
Trauma	29	8	37
Vascular	14	0	14
Intracranial hematoma	20	0	20
Others	23	17	40
Total	147	33	180

#### Outcome

The overall complication rate was 44%.

#### Frequency of complications in different categories of diagnosis

Out of 147 cranial and 33 spinal cases, 74 cranial and 6 spinal cases developed complications, respectively. Under six spinal cases developed complications, three each were from the neoplasm and trauma categories (Table 4).

**Table 4:** Frequency of postoperative complications in different diagnostic categories: Number of patients developing postoperative complications across different diagnostic groups.

Diagnosis	Complication		Total
	Yes	No	
Congenital	2	13	15
Neoplasm	35 (cranial: 32, spinal: 3)	19	54
Trauma	17 (cranial: 14, spinal: 3)	20	37
Vascular	8	6	14
Intracranial hematoma	9	11	20
Others	9	31	40
Total	80	100	180

#### Frequency of complications classified according to the Modified Clavien Dindo Grade

Out of 80 patients with post-operative complications, the maximum number of patients fell under Clavien-Dindo Grade Ib, i.e., 18, and the least number of patients had a CDG of IIIb. There were 8 mortalities (Table 5).

**Table 5:** Distribution of complications according to Modified Clavien–Dindo Grades: Frequency and percentage of postoperative complications stratified by MCD grade.

Modified Clavien Dindo Grade (MCD)	Frequency (percentage)	Grade
Ia	11 (6.1)	Mild MCDG: 29 (16.1)
Ib	18 (10.0)	
IIa	15 (8.3)	Moderate MCDG: 22 (12.2)
IIb	7 (3.9)	
IIIa	16 (8.9)	Severe MCDG: 29 (16.1)
IIIb	5 (2.8)	
IV	8 (4.4)	
Total	80 (44)	

#### Frequency of postoperative complications

Among various complications, 13/180 (7.2%) patients needed ventilator support in the ICU for respiratory failure, 12/180 (6.7%) developed seizures, 11/180 (6.1%) patients developed surgical site infection (SSI), 5/180 patients had facial nerve palsy, etc. (Table 6).

**Table 6:** Types and frequencies of postoperative complications: Specific postoperative complications encountered in the study with their respective frequencies.

Complications	Frequency	Percent
Arrhythmia	1	0.6
Bleeding	3	1.7
CSF leak	3	1.7
Death	8	4.4
Delirium	1	0.6
Diabetes insipidus	3	1.7
DVT	1	0.6
Facial nerve palsy	5	2.8
Hemiplegia	1	0.6
Hydrocephalus	1	0.6
Meningitis	1	0.6
Multiorgan failure	4	2.2
Pneumonia	1	0.6
Pseudomeningocele	1	0.6
Seizure	12	6.7
Shunt malposition	1	0.6
SSI	11	6.1
Transient focal neurologic deficit	8	4.4
UTI	1	0.6
Ventilator support	13	7.2

#### ICU LOS in days

**Figure 1.**
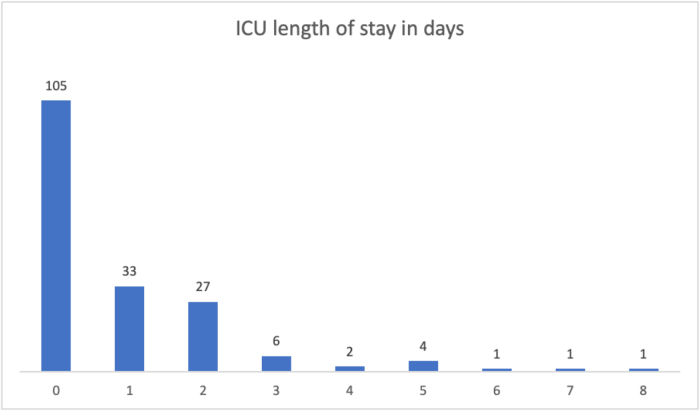
**ICU length of stay among neurosurgical patients**: Distribution of ICU stay duration in the study population.

Out of 180 patients, 105 did not require ICU admission, 33 required a 1-day ICU stay, and the maximum ICU stay was 8 days. The mean LOS in the ICU was 0.86 days (SD 1.387; Fig. 1).

#### Hospital LOS in days

**Figure 2.**
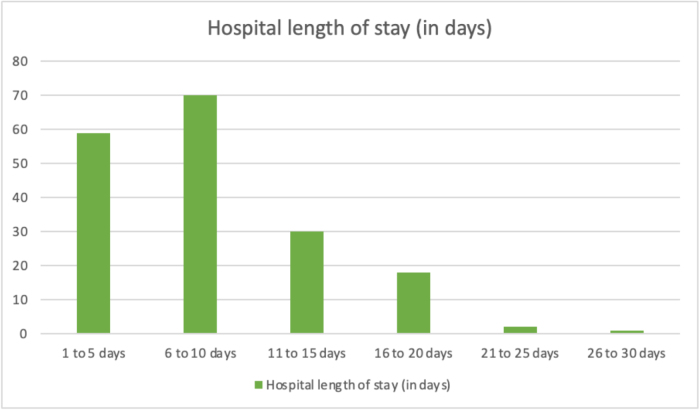
**Hospital length of stay among neurosurgical patients**: Distribution of total hospital stay durations.

The shortest hospital LOS was 1 day, and the longest was 30 days; the maximum number of patients stayed in the hospital for a duration between 6 and 10 days. The mean hospital LOS was 8.78 days (SD 5.256; Fig. 2).

#### Modified Rankin scale

**Figure 3.**
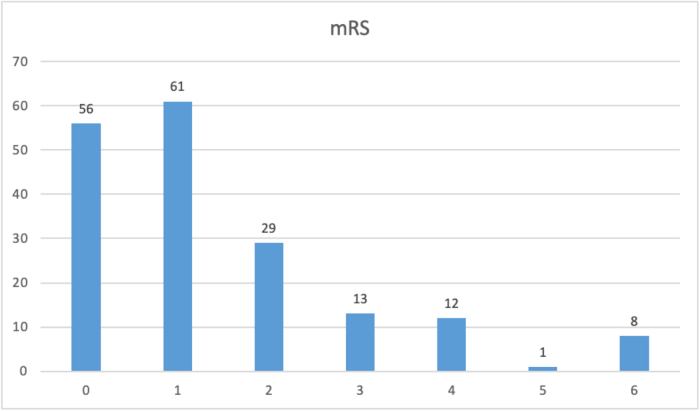
**Distribution of postoperative modified Rankin scale (mRS) scores**: Proportion of patients with good (mRS 0–3) and bad (mRS 4–6) outcomes.

Among 180 patients, 146 experienced good outcomes post-operatively, while the remaining 34 had bad outcomes (Fig. 3).

### Comparison of LOS, complications, and outcomes

The mean hospital LOS was significantly longer in patients with complications compared to those without (11.41 ± 6.0 vs 6.67 ± 3.2 days; independent-samples *t*-test, *P* < 0.05). The mean hospital LOS in mild MCDG was 11.38 (±4.3) days. The mean hospital LOS in moderate MCDG was 11 (±6.3) days. The mean hospital LOS in severe MCDG was 11.76 (±7.4) days (Tables 7 and 8).

**Table 7:** Hospital length of stay stratified by complication status: Distribution of total hospital length of stay among patients with and without complications.

		Complication		
		No	Yes	Total
Hospital length of of stay (days)	1	1	1	2
	2	0	3	3
	3	10	0	10
	4	20	4	24
	5	14	6	20
	6	12	7	19
	7	5	5	10
	8	16	6	22
	9	3	3	6
	10	9	4	13
	11	1	2	3
	12	5	8	13
	13	0	2	2
	14	2	5	7
	15	1	4	5
	16	0	3	3
	17	0	1	1
	18	0	5	5
	19	0	1	1
	20	1	7	8
	25	0	2	2
	30	0	1	1
Total		100	80	180

**Table 8:** Comparison of mean hospital length of stay in patients with and without complications: Mean hospital stay compared between patients with complications and those without using the independent-samples *t*-test.

	Complication	No complication	*P*-value
Mean hospital length of stay	11.41 (±6.0)	6.67 (±3.2)	<0.05

#### MCD grade and mRS comparison

**Figure 4.**
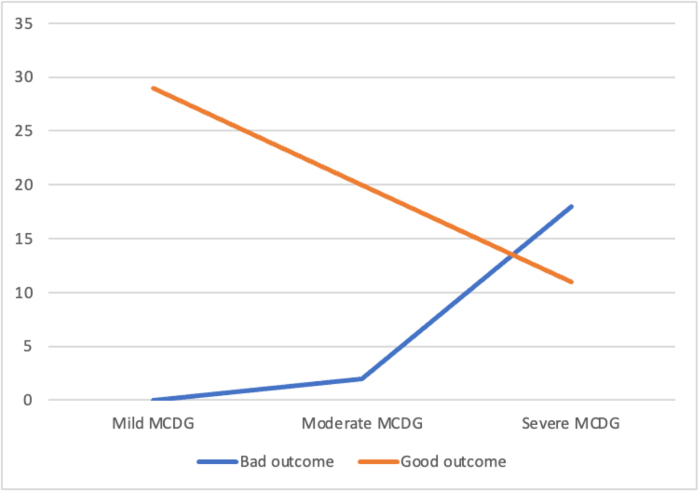
Relationship between MCD grade and mRS outcome: Trend of mRS outcomes across increasing MCD severity grades.

Table 9 shows the distribution of the patients across MCD and mRS scores. As the grade of MCD increases, the number of patients with bad outcomes also increases, with a *P*-value of <0.05, i.e., the correlation is statistically significant. Among 80 patients with complications, 60 had a good outcome, while the rest had a bad outcome (*P*-value of <0.05 Chi-square test), indicating statistical significance, as patients with bad outcomes were predominantly from severe MCDG (Table 10 and Fig. 4). The association of postoperative outcomes with complications, gender, comorbidities, types of surgery, and ICU stay are shown in the Table 11.

**Table 9:** Distribution of mRS scores across modified Clavien–Dindo Grades: Cross-tabulation of modified Rankin scale scores across various MCD grades.

MCD grade	mRS							Total
	0	1	2	3	4	5	6	
Ia	1	4	5	1	0	0	0	11
Ib	0	10	6	2	0	0	0	18
IIa	2	5	6	2	0	0	0	15
IIb	0	2	2	1	2	0	0	7
IIIa	0	0	3	6	7	0	0	16
IIIb	0	0	1	1	2	1	0	5
IV	0	0	0	0	0	0	8	8
No complication	53	40	6	0	1	0	0	100
Total	56	61	29	13	12	1	8	180

**Table 10:** Association between MCD severity and mRS outcome: Comparison of good and bad mRS outcomes across mild, moderate, and severe MCD categories using the chi-square test.

MCD grade	mRS Outcome		*P*-value
	Bad outcome	Good outcome	
Mild MCD	0	29	<0.05
Moderate MCD	2	20	
Severe MCD	18	11	
Total	20	60

**Table 11:** Comparison of clinical characteristics by mRS outcome: Association of postoperative outcomes with complications, gender, comorbidities, type of surgery, and ICU stay.

Characteristics	Total	Good outcome (mRS 0–3)	Bad outcome (mRS 4–6)	*P*-value
Complications	80	60	20	<0.01
Male gender	103	94	9	0.157
Presence of co-morbid conditions	34	29	5	0.556
Emergency surgery	68	57	11	0.142
Mean ICU length of stay	0.86 (±1.387)	0.52 (±0.81)	3.38 (±2.11)	<0.01

Demographics like gender, presence of co-morbidities, and type of surgery, whether elective or emergency, had no relation to outcome.

## Discussion

In today’s healthcare landscape, where patient safety and proper management are paramount, it is crucial to evaluate the anticipated outcomes of medical procedures. To achieve this, various scoring systems have been developed to predict risk-adjusted morbidity and mortality rates. Our study evaluated neurosurgical complications using the MCD grade system and assessed their correlation with patient outcomes as measured by the mRS. Neurosurgery is associated with high rates of mortality and morbidity due to the complexity of brain structures. The complications can range from mild postoperative nausea and vomiting to devastating neurological deterioration. Often, complications are analyzed in a specific group of patients. A large number of studies have been dedicated to reporting specific complications in specific subgroups of surgeries. However, it is essential to have an overview of all the complications that occur in a neurosurgical setting, which will aid in planning and executing measures required for the effective management of neurosurgical patients.

Fugate *et al*[5] reported a 14.3% complication rate in neurosurgery patients, with most cases being elective and only 6% being emergency cases. Houkin *et al*[10] reported 182 adverse events (28.3%) among 643 neurosurgical interventions over 2 years. Rolston *et al*[11], in their retrospective meta-analysis study, found that complications occurred in 14.3% (5507 of 38 396) of neurosurgical cases. Schiavolin *et al*[12] reported a complication rate of 22.6% among elective patients. Bosanto *et al*[13] reported the neurosurgical complication rate to be 16.36%. Landriel Ibanez *et al*[8] analyzed a cohort of 1190 patients and found that one or more complications occurred in 14% of patients (*n* = 167). Sarnthein *et al*[14] in their study included data from Zurich (and compared it simultaneously with Milano and Buenos Aires) using the CDC system (which is the basis of the Landriel scale) and reported a complication rate of 24% from a cohort of 1341 patients, most of them belonging to cranial surgery. In our study, complication rate was 44%. Compared to the other above-mentioned studies, the complication rate in our study was higher, which may be because we have included both elective [112 (62%)] and emergency surgeries [68 (38%)].

The rate of complications depends upon the “definition of complication,” the relative number of brain and spine cases, and the relative number of elective and emergency cases. Fugate *et al* reported that cranial procedures were 2.6 times more likely to produce complications (24%) compared to spinal procedures (11%)[5]. Rolston *et al* similarly noted that complications were far more likely to occur in cranial than spinal procedures (23.6% cranial vs. 11.2% spinal)[11]. In a study by Schiavolin *et al*, complications in cranial procedures were more frequent than spinal procedures (32.78 vs 7.04%); in our study, it was 12.3 times[12]. From the above data, overall complications will certainly depend upon the relative number of cranium and spine cases being operated on at a center. Hence, the reported higher complications are attributed to a larger number of cranial-related surgeries as seen in studies by Ibanez *et al*[8] (72% cranial and 28% spinal cases) and Schiavolin *et al* (60% cranial and 40% spinal cases). Similarly, our study had 82 and 18% of cranium and spine cases[12], respectively. Out of various categories of diagnosis, the maximum complications were seen in neoplasm and trauma cases. It may also be due to the relative number of cases in each category.

Out of 80 patients with postoperative complications, the maximum number of patients fell under Clavien Dindo grade Ib, i.e., 18, and the least number of patients had CDG of IIIb. There were 8 (4.4%) mortalities. In our study, the complication rates in the groups Ia, Ib, IIa, IIb, IIIa, IIIb, and IV were 6.1, 10.0, 8.3, 3.9, 8.9, 2.8, and 4.4%, respectively.

In a study by Vemula *et al*,[15] rates of complication in groups Ia, Ib, IIa, IIb, IIIa, IIIb, and IV were 19.8, 25.9, 14.2, 11.3, 9.9, 3.3, and 15.6%, respectively. Similarly, a study by Ibanez *et al*[8] study showed 13.16, 18.55, 10.17, 15.62, 17.96, 16.16, and 8.38%, respectively. A study by Schiavolin *et al*[12] showed 44.3, 16.66, 8.77, 18.89, 7.9, 0.88, and 2.6, respectively, and in Sarnthein *et al*[14], 22, 45, 4, 21, 5, 0, and 3% were the groups of complications. The most frequent grade of general complication in the Landriel Ibanez *et al*[8] study was Ib (18.55%), followed closely by IIIa (17.96%) and IIIb (16.16%), in the Schiavolin *et al*[12] study was Ia (44.3%), and in the Vemula *et al*[15] study was Ib (25.9%) followed by Ia (19.8%), and in our study was Ib (10%) followed by IIIa (8.9%).

The typical LOS for neurosurgical patients varies by diagnosis, procedure type, and patient comorbidities; however, several large cohort studies provide representative estimates.

The mean hospital LOS was 8.78 days, which is comparable to the median LOS of 8 days reported in a large European neurosurgical cohort[16] but slightly longer than the 6-day average reported by another large US series[17]. This difference may reflect variations in case mix, resource availability, and postoperative monitoring practices in our setting. The majority of our patients stayed 6–10 days, aligning with expected LOS patterns for general neurosurgical populations. Patients without complications had a significantly shorter LOS (mean 6.67 days), whereas those with complications stayed nearly twice as long (11.41 days), consistent with findings from other studies showing that postoperative morbidity substantially prolongs hospitalization.

Our ICU utilization was notably lower than the values reported[18]. The mean ICU LOS in our cohort was 0.86 days, which is far below the ICU stay described in neurosurgical ICU cohorts[19]. The proportion of patients requiring ventilatory support (7.2%) is also lower than rates reported in high-acuity neurosurgical populations, where prolonged ventilation contributes substantially to ICU LOS[20]. These differences likely reflect the predominance of less complex neurosurgical procedures in our study and effective postoperative triage practices.

In a study by Sebok *et al*[21], postoperative complications were classified as CD grade, and the outcome was measured using the mRS. A significant correlation was found between CD grade > I and an increase in mRS score and hospital LOS. In our study, a significant correlation was also observed between MCD grade and mRS and hospital LOS. The overall mortality rate was 1.17% in a study by Landriel Ibanez *et al* study[8], 0.6% in a study by Schiavolin et al[12], 3.3% in a study by Houkin *et al* [10], and 2.56% in a study by Chandra Venkata Vemula *et al*[15]. Our study reported a mortality rate of 4.4%.

### Limitations

One of the main strengths of our study is its prospective design and the use of validated grading systems, which enhance the generalizability and reliability of our findings. However, limitations include a single-center setting and a relatively small sample size, which may limit the external validity. Another limitation of our study was the lack of long-term follow-up for post-operative complications. Future multicenter studies with larger cohorts could provide a more comprehensive understanding of the interplay between complication severity and patient functional outcomes.

## Conclusion

The overall postoperative complication rate was 44%, with the highest rate of complications in MCD grade Ib (10%). Patients with a severe grade of complications had bad mRS outcomes, and patients with complications had longer hospital stays. There was a significant correlation of MCD Grade with both mRS outcome and hospital LOS, with a *P* value of <0.05. Proper stratification of postoperative complications in neurosurgery helps to predict postoperative outcomes in patients. The MCD Grade is a predictive tool used to measure postoperative outcomes according to the mRS in neurosurgical patients.

## Data Availability

All the data generated during this study can be accessed through direct communication with the corresponding author and the agreement of all research team members.
